# Cordycepin Decreases Compound Action Potential Conduction of Frog Sciatic Nerve In Vitro Involving Ca^**2+**^-Dependent Mechanisms

**DOI:** 10.1155/2015/927817

**Published:** 2015-05-19

**Authors:** Li-Hua Yao, Hui-Min Yu, Qiu-Ping Xiong, Wei Sun, Yan-Liang Xu, Wei Meng, Yu-Ping Li, Xin-Ping Liu, Chun-Hua Yuan

**Affiliations:** ^1^School of Life Science, Jiangxi Science & Technology Normal University, Nanchang, Jiangxi 330013, China; ^2^Department of Pathogenic Biology & Immunology, Medical College, Shenzhen University, Shenzhen, Guangdong 518060, China; ^3^Internal Medicine Department 3, Jiangxi Province Tumor Hospital, Nanchang, Jiangxi 330029, China; ^4^Internal Medicine Department 2, Jiangxi Province Tumor Hospital, Nanchang, Jiangxi 330029, China

## Abstract

Cordycepin has been widely used in oriental countries to maintain health and improve physical performance. Compound nerve action potential (CNAP), which is critical in signal conduction in the peripheral nervous system, is necessary to regulate physical performance, including motor system physiological and pathological processes. Therefore, regulatory effects of cordycepin on CNAP conduction should be elucidated. In this study, the conduction ability of CNAP in isolated frog sciatic nerves was investigated. Results revealed that cordycepin significantly decreased CNAP amplitude and conductive velocity in a reversible and concentration-dependent manner. At 50 mg/L cordycepin, CNAP amplitude and conductive velocity decreased by 62.18 ± 8.06% and 57.34% ± 6.14% compared with the control amplitude and conductive velocity, respectively. However, the depressive action of cordycepin on amplitude and conductive velocity was not observed in Ca^2+^-free medium or in the presence of Ca^2+^ channel blockers (CdCl_2_/LaCl_3_). Pretreatment with L-type Ca^2+^ channel antagonist (nifedipine/deltiazem) also blocked cordycepin-induced responses; by contrast, T-type and P-type Ca^2+^ channel antagonists (Ni^2+^) failed to block such responses. Therefore, cordycepin decreased the conduction ability of CNAP in isolated frog sciatic nerves via L-type Ca^2+^ channel-dependent mechanism.

## 1. Introduction

*Cordyceps militaris* is a rare but renowned caterpillar fungus used as traditional Chinese medicine. This fungus has been widely utilized in oriental countries as tonic to prevent early aging, improve physical performance, and increase lifespan [1–3]. Cordycepin (3-deoxyadenosine), a major component of* C. militaris*, was first isolated from the ascomycete fungus* C*.* militaris *[4]. Cordycepin exhibits various biological properties, such as antitumor [5], anti-inflammatory [6], antioxidation [4], and anti-diabetic [7]. Thus, cordycepin and cordycepin-related substances have been considered as well-known healthcare products, particularly in the oriental countries [1–8]. Cordycepin may also modulate the function of the central nervous system (CNS) [2, 4, 8–10]. Cordycepin elicits significant neuroprotective effects against damage caused by ischemia/reperfusion injury; these neuroprotective effects occur because cordycepin performs free radical scavenging activity and prevents neuronal cell death [4, 9]. Cordycepin can decrease neuronal activity through membrane hyperpolarization and suppress synaptic transmission via presynaptic mechanisms [2, 8]. Therefore, cordycepin is an important mediator in the modulation of brain pathological and physiological processes.

Peripheral nervous system (PNS), another important part of the nervous system, is distributed all over the body and links the CNS comprising the brain and the spinal cord to other organs of the body; thus, the CNS can react to changes in internal and external environments via afferent nerves transmitting sensory information and regulate body functions, including physical performance via efferent nerves conveying motor information, to ensure the integrity and unity of the human body to adapt to changes in the external environment [11–14]. However, the regulatory effect of cordycepin on the PNS remains unclear. Further studies are necessary to address these pharmacological differences.

Sciatic nerve is the largest nerve of the PNS frequently used to investigate processes associated with PNS [14–17]. Compound nerve action potential (CNAP), the basic component of nerve activity, is necessary to regulate physical performances, including motor system physiological and pathological processes; CNAP is also implicated in signal conduction in PNS [14–17]. Therefore, this study was conducted to investigate the regulatory effect of cordycepin on CNAP conduction ability of sciatic nerve in vitro and the possible mechanisms of cordycepin action.

## 2. Material and Methods

### 2.1. Drug Preparation

Chemicals used for making Ringer's solution (RS) were purchased from Sigma Co. (St. Louis, MO, USA). Frog's RS provides the equivalent physiologic condition of the frog and is composed of 112 mM NaCl, 2 mM KCl, 1.5 mM CaCl_2_, 0.1 mM NaH_2_PO_4_, and 2.38 mM NaHCO_3_ [3]. The pH of the RS was adjusted to 7.2, and all measurements were recorded with the preparations equilibrated at room temperature (22–25°C). Cordycepin with a purity of more than 98% was provided by the South China Normal University [8, 18]. Cordycepin was dissolved in RS at concentrations of 0, 20, 50, 100, and 200 mg/L.

### 2.2. Tissue Preparation

The care and use of animals and the experimental protocol of this study were approved by the Institutional Care and Use Committee of our university. Frogs were euthanized by decapitation and destruction of the spinal brain [8]. Sciatic nerve was dissected out and placed in a Petri dish containing frog RS. The connective tissue and sheath surrounding the nerve were removed carefully. A bundle containing a small group of nerve fibres was then allowed to equilibrate in bath conditions for 30 min. The nerve fibres were immersed in the RS or dropped continuously with RS during preparation or throughout the recordings.

In separate experiments with a Ca^2+^-free medium, the nerve fibres were immersed or dropped with Ca^2+^-free RS during the preparation or throughout the recordings [17]. In another set of experiments, the nerve fibres were immersed or dropped with the RS supplemented with Ca^2+^ channel blockers CdCl_2_ (0.4 mM); LaCl_3_ (0.15 mM); nifedipine (10 *μ*M); deltiazem (10 *μ*M); and NiCl_2_ (0.3 mM), respectively, during preparation or throughout the recordings.

### 2.3. Electrophysiological Techniques

**Figure 1:**
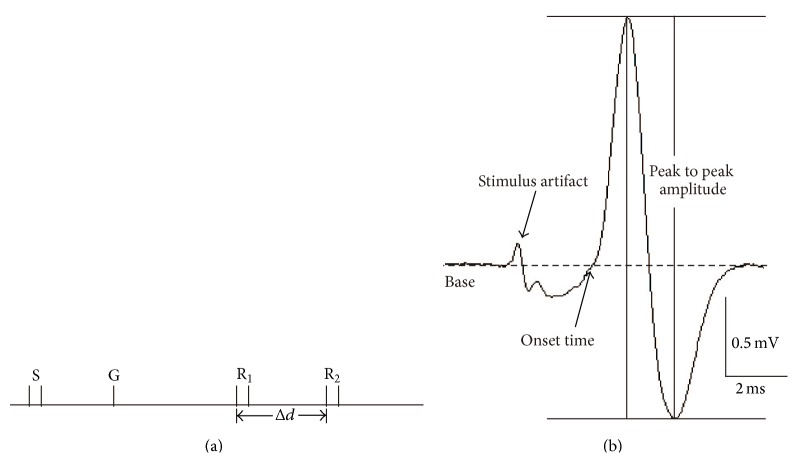
CNAP determination. (a) Set of the 7 Ag/AgCl electrodes, including S (1 pair of stimulating electrodes), G (1 grounding electrode), and R1 and R2 (2 pairs of recording electrodes). Δ*d* is the space between the electrodes of R1 and R2, which were fixed during the whole procedure. (b) A normal sample trace of CNAP without drug exposure. The time points of the onset time, peak to peak amplitude are shown.

CNAPs were recorded using an extracellular recording technique with a PowerLab data acquisition system (ADInstruments, Inc., Colorado Springs, CO, USA) [3, 15]. After 30 min of stabilization in RS, segments of nerve measuring 5 cm were placed in a Plexiglas nerve chamber containing 7 Ag/AgCl electrodes including 1 pair of stimulating electrodes, 1 grounding electrode, and 2 pairs of recording electrodes. The space between the electrodes was fixed during the whole procedure (Figure 1(a)). The stimulating voltage was set to produce a maximal CNAP using single square pulses of supramaximal strength and 0.1 ms in duration.

### 2.4. CNAP Determination

CNAP recording experiments were performed as described previously [15]. CNAP recorded from each nerve trunk before exposure to cordycepin was considered the control. Cordycepin with different concentration in RS was continuously and gently dropped on the nerves between the first pair of recording electrodes at a speed of 30 mL/h for 5 min as the maximum effect was seen at 1 min, and CNAPs were recorded every 30 s. The nerves were then washed continuously with RS at a speed of 30 mL/h for 10 min, and CNAPs were recorded again every 30 s.

CNAP parameters mainly include the amplitude (defined as the height in mV from the peak of the positive phase to the peak of the negative phase; Figure 1(b)) and the conductive velocity. Conductive velocity was calculated as follows:

(1) $$\begin{array}{c} \text{Conductive velocity}=\frac{\Delta d}{\Delta T}, \end{array}$$

where Δ*d* is the space between the 2 pairs of recording electrodes, which were fixed during the whole procedure (Figure 1(a)) and Δ*T* is the time point between the onset time points of CNAP recording from each pair of recording electrodes (Figure 1(b)). In general, decrease of these parameters represents a reduction of nerve conduction abilities [15, 19].

### 2.5. Data Analysis

The same subject was observed using a repeated design. In this design, each subject served as the corresponding control sample (self-reference). The mean of individual CNAP amplitudes recorded under stable conditions at the start of the experiment was considered as baseline, and the amplitudes obtained after cordycepin was applied were expressed as percentage of the baseline to characterize the effects of cordycepin on nerve conduction ability. To study the effect of cordycepin on the CNAP conductive velocity, the mean velocity of the stable-state before exposure to cordycepin was considered as the control value and scaled to 100%. The mean velocity of the stable-state in the presence of cordycepin was normalized to the corresponding values before cordycepin was applied.

Numerical data were presented as mean ± SEM unless otherwise indicated. Significant difference was calculated using Student's paired *t*-test. *P* < 0.05 indicated statistical significance.

## 3. Results

### 3.1. Cordycepin Decreased the Amplitude and Conductive Velocity of CNAP

**Figure 2:**
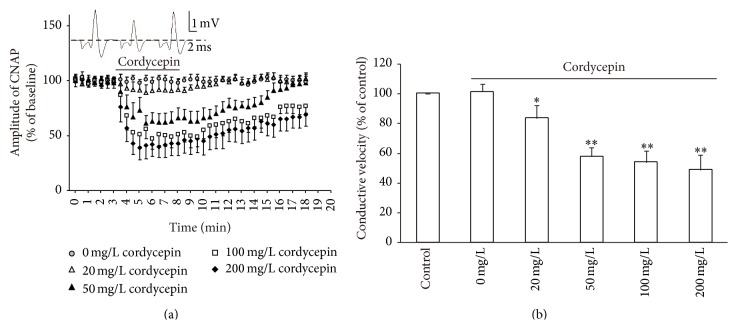
Effects of cordycepin on amplitude and conductive velocity of compound nerve action potential (CNAP) in isolated frog sciatic nerve. (a) Sample traces (top) show the CNAP recorded before, during, and after 50 mg/L cordycepin was applied. Below are the time courses of the effects of different concentrations of cordycepin (0, 20, 50, 100, and 200 mg/L) on the amplitude of CNAP conduction. Horizontal bar indicates the bath application of cordycepin. (b) Summary of changes in conductive velocity of CNAP induced by different concentrations of cordycepin. The velocity of the stable state before exposure to cordycepin is defined as the control value and scaled to 100%. ^*∗*^ *P* < 0.05, ^*∗∗*^ *P* < 0.01 compared with the control group.

Figure 2 illustrates the effects of cordycepin on the CNAP conduction in isolated frog sciatic nerve. Cordycepin treatment profoundly depressed the amplitude of CNAP in a concentration-dependent manner. Cordycepin-mediated decrease in CNAP occurred within 1 min after cordycepin was applied. This decrease peaked as the stable state at 2–4 min. By contrast, the amplitude of the normal RS group (0 mg/L cordycepin application) remained stable throughout the experiment. After 3–10 min of washout with RS, the amplitude recorded at different cordycepin concentrations gradually returned to baseline level. At 20, 50, 100, and 200 mg/L, the resultant percentages of CNAP amplitudes were decreased to 91.3 ± 8.92% (*n* = 13), 62.18 ± 8.06% (*n* = 15), 50.26 ± 9.87% (*n* = 12), and 41.57 ± 11.43% (*n* = 8; Figure 2(a)). The corresponding recovery time to the baseline level after washout were 1-2, 3–6, >10, and >10 min. After cordycepin application, the conductive velocity was lower than that before the drug exposure. Particularly in the 50 mg/L group, the conductive velocity of CNAP was very significantly decreased (57.34% ± 6.14% of control, *n* = 15, *P* < 0.01). After cordycepin was increased to 100 and 200 mg/L, the depressive effect of this treatment on velocity increased correspondingly; however, conductive velocity at 50 mg/L was not significantly different from that at 100 and 200 mg/L cordycepin (*P* > 0.05; Figure 2(b)). These results revealed that cordycepin may elicit a regulatory effect on signal conduction in PNS by decreasing CNAP conduction ability; such effects of cordycepin are reversible.

At 50 mg/L cordycepin, amplitude and conductive velocity of CNAP significantly decreased (*P* < 0.01) and recovered quickly after washout occurred; thus, this concentration was used for subsequent tests.

### 3.2. Cordycepin-Induced Decrease in Amplitude and Conductive Velocity Were Absent in Ca^2+^-Free Medium

**Figure 3:**
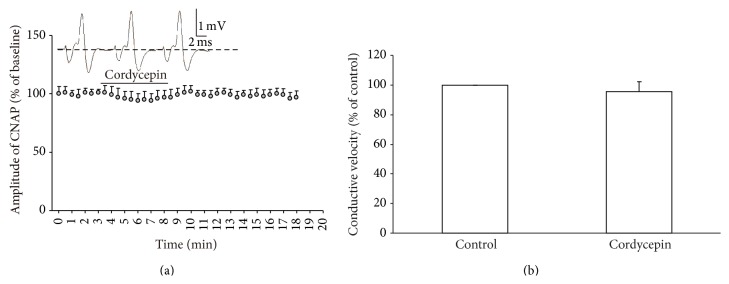
Effects of 50 mg/L cordycepin on amplitude and conductive velocity of compound nerve action potential (CNAP) in Ca^2+^-free medium. (a) Sample traces (top) show the CNAP recorded before, during, and after cordycepin was applied. Below are the time courses of the effects of cordycepin on the amplitude of CNAP conduction. Horizontal bar indicates the bath application of cordycepin. (b) Summary of changes in conductive velocity of CNAP induced by cordycepin. The velocity of the stable state before the nerve was exposed to cordycepin is defined as the control value and scaled to 100%.

Ca^2+^ signals perform critical functions in signal conduction in PNS [20, 21]. A decrease in Ca^2+^ signals is generally coupled with a decrease in CNAP conduction ability [17, 21]. Thus, the effect of cordycepin on signal conduction was investigated using the same stimulation procedure as aforementioned (Figure 2) in the Ca^2+^-free medium. After control recordings were performed, the prepared nerve was exposed to cordycepin for 5 min (Figure 3). Our result showed that amplitude was not altered (94.16% ± 5.82% control, *n* = 16, *P* > 0.05) before and after cordycepin was applied (Figure 3(a)). Furthermore, cordycepin-induced decrease in conductive velocity was blocked in the Ca^2+^-free medium (95.38 ± 6.91% of the control group, *n* = 16, *P* > 0.05; Figure 3(b)). These results indicate the novel role of the regulatory effect of cordycepin on signal conduction in PNS involving Ca^2+^-dependent mechanisms.

### 3.3. CdCl_2_/LaCl_3_ Blocked the Effect of Cordycepin-Induced Decrease in Amplitude and Conductive Velocity

Extracellular Ca^2+^ influxes through Ca^2+^ channels are crucial mechanisms that produce Ca^2+^ signals [3, 10, 22]. Therefore, the effects of cordycepin on CNAP conduction in the isolated sciatic nerve were investigated using RS supplemented with 0.4 mM CdCl_2_ or 0.15 mM LaCl_3_ to block all Ca^2+^ channels [3, 22]. Similar to the results shown in Figure 3, the amplitude and conductive velocity were not altered in the presence of cordycepin; this result was observed not only in RS supplemented with CdCl_2_ medium but also in RS supplemented with LaCl_3_ medium. Indeed, cordycepin decreased the amplitude and conductive velocity of CNAP in the isolated sciatic nerve involving Ca^2+^-dependent mechanisms; extracellular Ca^2+^ influxes through Ca^2+^ channels are strongly recommended.

### 3.4. Nifedipine/Deltiazem Blocked the Effect of Cordycepin-Induced Decrease in Amplitude and Conductive Velocity

L-type Ca^2+^ channel, as a typical Ca^2+^ channel, is present in nerve fibers and involved in neural activity [17, 23]. We subsequently investigated the effect of cordycepin on CNAP in RS supplemented with 10 *μ*M nifedipine or 10 *μ*M deltiazem to block L-type Ca^2+^ channel [17]. Cordycepin could not induce significant changes in the amplitude and conductive velocity of CNAP in RS supplemented with nifedipine medium or in the RS supplemented with deltiazem medium (Figure 5). These results indicate that the mechanism of Ca^2+^ influxes through L-type Ca^2+^ channel is involved in the regulatory effect of cordycepin on CNAP conduction in the isolated sciatic nerve.

### 3.5. NiCl_2_ Failed to Block the Effect of Cordycepin-Induced Decrease in Amplitude and Conductive Velocity

To further clarify the mechanism by which cordycepin modulates CNAP conduction in PNS, we detected the effect of cordycepin on CNAP in RS supplemented with 0.3 mM NiCl_2_, T-type and P-type Ca^2+^ channel antagonist. NiCl_2_ failed to block the cordycepin-induced decrease in amplitude and conduction velocity of CNAP in the isolated sciatic nerve (Figure 6). Cordycepin induced a decrease in amplitude within 1 min. This decrease peaked as a stable-state at 2–4 min. After 3–10 min of washout, the amplitude recorded in the cordycepin-treated groups gradually returned to the baseline level. The resultant percentages of CNAP amplitudes were decreased to 68.19 ± 7.11% (*n* = 14; Figure 6(a)), which is very significant compared with the baseline level (*P* < 0.01). After cordycepin was applied, conductive velocity after cordycepin was applied was lower than conductive velocity before cordycepin was applied (Figure 6(b); *P* < 0.01). Consistent with the result shown in Figure 2, these results indicated that T-type and P-type Ca^2+^ channels are not involved in the regulatory effect of cordycepin on CNAP conduction in isolated sciatic nerves.

## 4. Discussion

**Figure 4:**
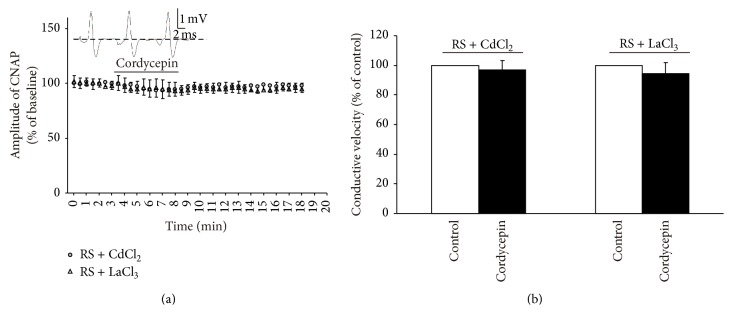
Effects of 50 mg/L cordycepin on amplitude and conductive velocity of compound nerve action potential (CNAP) in RS supplemented with 0.4 mM CdCl_2 _or 0.15 mM LaCl_3_. (a) Sample traces (top) show the CNAP recorded before, during, and after cordycepin was applied in RS + CdCl_2 _medium. Below are the time courses of the effects of cordycepin on the amplitude of CNAP conduction. Horizontal bar indicates the bath application of cordycepin. (b) Summary of changes in conductive velocity of CNAP induced by cordycepin. The velocity of the stable state before the nerve was exposed to cordycepin is defined as the control value and scaled to 100%.

**Figure 5:**
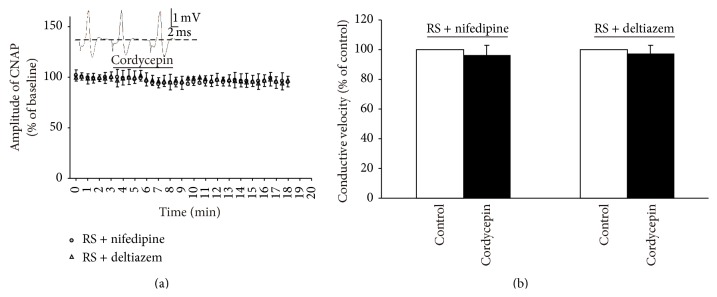
Effects of 50 mg/L cordycepin on amplitude and conductive velocity of compound nerve action potential (CNAP) in RS supplemented with 10 *μ*M nifedipine or 10 *μ*M deltiazem. (a) Sample traces (top) show the CNAP recorded before, during, and after cordycepin was applied in RS + nifedipine medium. Below are the time courses of the effects of cordycepin on the amplitude of CNAP conduction. Horizontal bar indicates the bath application of cordycepin. (b) Summary of changes in conductive velocity of CNAP induced by cordycepin. The velocity of the stable state before the nerve was exposed to cordycepin is defined as the control value and scaled to 100%.

**Figure 6:**
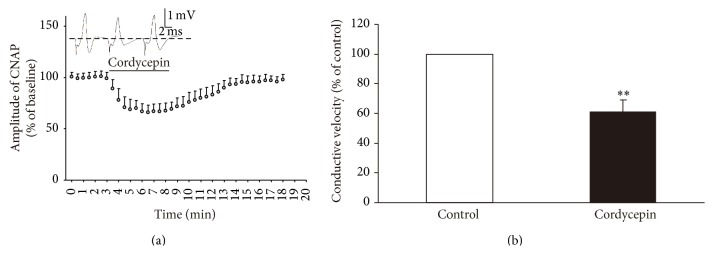
Effects of 50 mg/L cordycepin on amplitude and conductive velocity of compound nerve action potential (CNAP) in RS supplemented with 0.3 mM NiCl_2_. (a) Sample traces (top) show the CNAP recorded before, during, and after cordycepin. Below are the time courses of the effects of cordycepin on the amplitude of CNAP conduction. Horizontal bar indicates the bath application of cordycepin. (b) Summary of changes in conductive velocity of CNAP induced by cordycepin. The velocity of the stable state before the nerve was exposed to cordycepin is defined as the control value and scaled to 100%. ^*∗*^ *P* < 0.05, ^*∗∗*^ *P* < 0.01 compared with the control group.

In this study, cordycepin decreased the amplitude and conductive velocity of CNAP in the isolated frog sciatic nerve (Figure 2). In general, a decrease in amplitude and conductive velocity corresponds to the reduction of nerve conduction abilities [15, 19]. Thus, these results implied that the nerve conduction abilities were decreased by cordycepin. Cordycepin-induced decrease in amplitude and conductive velocity was blocked in the Ca^2+^-free medium or in the presence of all Ca^2+^ channel blockers (0.4 mM CdCl_2_ or 0.15 mM LaCl_3_; Figures 3 and 4) [3, 22]. L-type Ca^2+^ channel antagonist (nifedipine/deltiazem) also blocked the depressive action of cordycepin on CNAP (Figure 5). By contrast, T-type and P-type Ca^2+^ channel antagonist (NiCl_2_) failed to block the cordycepin-induced depressive action on CNAP (Figure 6). Therefore, cordycepin can modulate the nerve conduction abilities by decreasing the amplitude and conductive velocity of the CNAP; the mechanisms involving Ca^2+^ influxes through L-type Ca^2+^ channel are strongly recommended.

Changes in nerve conduction abilities were biologically significant. Nerve conduction abilities are used as a physiological/pathological index to identify nerve activity in signal conduction [11–17], which is necessary to regulate physical performance, including motor system physiological and pathological processes [14, 20, 23]. In the PNS, decreased neural activity usually has been used to decrease local response sensitivity to external stimulation, which contributes to reduce local metabolic rate and analgesia [14, 15, 17]. Interestingly, our current study demonstrates that cordycepin can depress neural conduction abilities by decreasing the amplitude and conductive velocity of CNAP; this result indicated that cordycepin can be used to modulate local physiological/pathological status. In our previous study, 50 mg/L of cordycepin significantly improves the physical fitness of skeletal muscles by decreasing muscle contractile response sensitivity to external stimulation; as a result, the antifatigue ability of the motor system is improved [3]. Combined with our previous finding, our current result provides further insights into the depressive action of cordycepin in CNAP conduction as an important mechanism involved in the affections of motor system physiological and pathological processes [3].

Ca^2+^ plays an important role in CNAP conduction [17, 20, 21]; L-type Ca^2+^ channels are present in nerve fibers and involved in neural activity [17, 23]. Our observations in Ca^2+^ channel antagonists revealed that cordycepin-induced reduction responses on nerve conduction abilities are mediated through Ca^2+^ influx via L-type Ca^2+^ channels. Action potential is determined by Na^+^ inward current and K^+^ outward current [19]. A decrease in Na^+^ inward current or an increase in K^+^ outward current likely reduces the amplitude of action potential and/or the rate of depolarization; as a result, CNAP amplitude and mean conduction velocity are affected [17, 19]. Therefore, Ca^2+^-dependent mechanism of the depressive effect of cordycepin on nerve conduction abilities may be related to a decrease in Na^+^ inward current and/or an increase in K^+^ outward current through Na^+^/Ca^2+^ exchangers and/or Ca^2+^-activated K^+^ channel [17, 24], respectively. Further studies should be conducted to clarify this possibility.

To our knowledge, this study is the first to show direct evidence of the regulatory effects of cordycepin on nerve conduction abilities in an isolated frog sciatic nerve. Our results showed that cordycepin can modulate nerve conduction abilities by decreasing the amplitude and conductive velocity of CNAP; mechanisms involving Ca^2+^ influxes through L-type Ca^2+^ channel are strongly recommended. Therefore, cordycepin can regulate local physical performances, including motor system physiological and pathological processes.
